# Challenges for health systems in Latin America: far beyond emergencies

**DOI:** 10.1590/0102-311XEN112826

**Published:** 2026-08-03

**Authors:** Cristiani Vieira Machado, Mónica Uribe-Gómez, Alex Alarcón Hein

**Affiliations:** 1 Escola Nacional de Saúde Pública Sergio Arouca, Fundação Oswaldo Cruz, Fiocruz, Rio de Janeiro, Brasil.; 2 Facultad de Ciencias Humanas y Económicas, Universidad Nacional de Colombia, Medellín, Colombia.; 3 Escuela de Salud Pública Salvador Allende, Universidad de Chile, Santiago, Chile.

The COVID-19 pandemic was one of the largest health emergencies ever recorded, with over 7 million confirmed deaths worldwide and total deaths estimated at around 20 million, accounting for underreporting and overall excess mortality. Additionally, negative repercussions on health care for other diseases, resulting from an overburdened health system, led to deaths from various causes and had medium- and long-term consequences. The multidimensional crisis associated with the health emergency was brutal, highlighting the perverse relation between the pandemic and global and national inequalities ^1^.

Six years after the pandemic, uncertainties and challenges remain concerning scientific knowledge and health policies, within a scenario of changing world geopolitics, threats to countries’ sovereignty, and setbacks in social and human rights.

The pandemic has highlighted the limitations of multilateralism, mechanisms for integration between countries, and the responses of national health systems worldwide, requiring significant changes on several fronts. Globally, it spurred debate on preparedness for future health emergencies and led to the signing of the Pandemic Agreement after over three years of negotiations, the implementation of which depend on supplementary regulations ^2^. 

Regarding national health systems, preparedness for identifying and responding to health emergencies has also been prominent on the agenda, with emphasis on information systems, monitoring and alert systems, surveillance and identification of pathogens, strengthening laboratory systems and, in some cases, the issue of vaccines. Furthermore, the analyses and proposals focused heavily on the “resilience” of these systems, with a variety of conceptions of what it means and its methodological, practical, and policy implications ^3^. However, even comprehensive definitions of resilience seem insufficient to account for the problems highlighted, many of which predate the crisis scenario, extend beyond the scope of emergencies, and transcend national borders.

Latin America has been severely impacted by the multidimensional crisis stemming from COVID-19, with profound economic, social, and health repercussions ^4^. The region reflects the complex interaction between structural, institutional, and political factors in the ways countries have responded and in the tragic recorded outcomes. Structural inequalities, associated with dependent economies, high levels of informal employment, and weak social protection systems, created an adverse context for dealing with a complex crisis.

Most Latin American health systems have historically been characterized by client segmentation, organizational fragmentation, public underfunding, and State subsidies to the private sector, which tends to concentrate in capital cities and economically dynamic areas. In recent decades, several countries have implemented health reforms with different meanings, scopes, and timing. In some cases, changes in the institutional configuration of health systems did not overcome their structural contradictions.

Brazil’s Health Reform in the 1980s, in the context of democratization, led to the creation of the Unified National Health System (SUS, acronym in Portuguese), a public and universal system whose implementation in the following decades fostered important advances in health, albeit with insufficient funding and without conflicting with the interests of the private sector in the country. Chile and Colombia adopted privatizing reforms in the 1980s and 1990s that reinforced segmentation and inequalities between social groups, with subsequent initiatives for incremental changes aimed at mitigating inequities. During this period, Argentina maintained a segmented, labor-based healthcare system, with the progressive intensification of market mechanisms and the expansion or contraction of specific public programs, depending on government’s orientation. Mexico, without breaking with the prevailing labor-based Social Security arrangement, adopted a Popular Health Insurance program between 2000 and 2018 to expand coverage for informal workers and the poor, with questionable results. Changes in administration altered the course of universalization strategies, and in 2026, the creation of a Universal Health Service was announced.

These health systems, which were already struggling to meet health needs in the face of demographic, epidemiological, and social changes, had difficulty in coordinating their response to the COVID-19 pandemic due to internal limitations and global issues. Global asymmetries in scientific and technological development, in the production and access to strategic supplies (such as vaccines), and dependence on imports have exacerbated the vulnerability of Latin American nations. During the pandemic, countries sought various solutions, which involved bilateral negotiations and agreements with the transnational pharmaceutical industry for purchasing supplies and, in a few cases like in Brazil, for transferring technologies. 

In some countries, the political landscape was also adverse: neoliberal, conservative, and anti-democratic governments weakened public institutions and hampered their ability to respond by omission, denialism, or limited social dialogue. The experience of Latin American countries in recent decades reveals that politics and government leadership matter as much in periods of relative stability as in times of crisis. 

Addressing the challenges of post-pandemic health systems in Latin America, therefore, requires more than preparedness to respond to health emergencies − undoubtedly an essential role of the state in public health − or even the resilience of health systems. It requires understanding and addressing their structural problems in the areas of financing, science and technology, the Health Economic-Industrial Complex (HEIC), public-private relations, labor management, professional training, and health care models. This includes continuously strengthening primary health care and health surveillance with capacities related to identifying and responding to emergencies. It also requires a strong commitment to universalization strategies, seeking to overcome barriers associated with labor market participation or the ability to pay for services, and to build solid institutional foundations for universal access. Also essential is to coordinate with other public policies, considering the social determinants of health and disease, combined with epidemiological knowledge and understanding of social dynamics. 

In other words, well-structured, funded, and integrated public health systems, with institutional strength in their various dimensions, will be better prepared to meet the daily health needs of populations and to identify and respond to health emergencies in a more timely and appropriate manner.

Finally, facing these challenges, including their technical and scientific dimension, also involves both a national and international political dimension. In the global scenario, the rise of far-right governments, the intensification of conflicts, and recent geopolitical changes − including the United States’ withdrawal from the World Health Organization ^5^ ^,^ ^6^ affect multilateralism and impact countries ^7^. Latin America needs to strengthen international cooperation and regional integration in foreign policy ^8^, as well as to promote other articulation initiatives in the Global South to increase health sovereignty ^9^. In some Latin American countries, economic and political instability are a cause for concern, as are neoliberal, conservative, and anti-democratic governments, which threaten hard-won social rights and hinder progress in healthcare. However, different groups within society must remain vigilant regarding government initiatives in the region. Any changes in administration, regardless of its ideology or agenda, should not infringe upon rights already acquired: it is an ethical duty to recognize progress, and to criticize setbacks.

This complex scenario requires research and critical thinking about on the role of institutions and political agency in reshaping policy trajectories and building more egalitarian societies in Latin America. The region has historically been a place of exploitation, violence, and injustice; but also of resistance, struggle, and reconstruction. Expanding rights, including in ​​healthcare, has always involved the mobilization of diverse social actors, often in adverse contexts. National health systems, if strengthened in their public and universal character, can do much more than respond to emergencies: as pillars of inclusive development, social protection, skilled work, and democratic participation, they offer broad possibilities for practical action, with immense potential for social transformation and impact on the living conditions of peoples.

By bringing together analyses of different trajectories and strategic issues to be faced by health systems in the region, the Supplement *Challenges of Health Systems in Post-Pandemic Latin America* of *Cadernos de Saúde Pública* expands and materializes the agenda proposed in this editorial, offering insights into paths for institutional strengthening, regional cooperation, and affirmation of the right to health. Its contributions, therefore, deepen the debate and support a perspective of health system transformation guided by equity, universality, and health sovereignty.
