# Supplementary material for: Challenges for health systems in Latin America: far beyond emergencies
Source: Cad Saude Publica. 2026 Aug 3;42(Suppl 1):e00112826. doi: 10.1590/0102-311XEN112826 (PMC13435630; doi:10.1590/0102-311XEN112826)
Supplement: Translation

# Translation: Desafios dos sistemas de saúde na América Latina: muito além das emergências

**DOI:** 10.1590/0102-311XPT112826

**Authors:** Cristiani Vieira Machado, Mónica Uribe-Gómez, Alex Alarcón Hein

**Affiliations:** 1 Escola Nacional de Saúde Pública Sergio Arouca, Fundação Oswaldo Cruz, Rio de Janeiro, Brasil.; 2 Facultad de Ciencias Humanas y Económicas, Universidad Nacional de Colombia, Medellín, Colombia.; 3 Escuela de Salud Pública Salvador Allende, Universidad de Chile, Santiago, Chile.

A pandemia de COVID-19 foi uma das maiores emergências sanitárias já registradas, com mais de 7 milhões de mortes confirmadas no mundo e estimativas de cerca de 20 milhões de óbitos totais causados pela emergência, considerando a subnotificação de casos e o excesso de mortalidade geral. Acrescente-se a repercussão negativa na atenção a outras doenças, decorrente da sobrecarga do sistema de saúde, que gerou mortes por diversas causas e consequências a médio e a longo prazo. A crise multidimensional associada à emergência sanitária foi brutal, evidenciando a perversa relação entre a pandemia e as desigualdades globais e internas dos países ^1^.

Passados seis anos do início da pandemia, permanecem incertezas e desafios nas esferas do conhecimento científico e das políticas de saúde, em um cenário de mudanças na geopolítica mundial, de ameaças à soberania dos países e de retrocessos nos direitos sociais e humanos.

A pandemia evidenciou os limites do multilateralismo, dos mecanismos de integração entre países e das respostas dos sistemas nacionais de saúde em todo o mundo, o que exige mudanças contundentes em diversos planos. Globalmente, impulsionou o debate sobre a preparação para futuras emergências sanitárias e levou à celebração do Acordo sobre Pandemias, após mais de três anos de negociações, cuja implantação depende de normas complementares ^2^. 

Quanto aos sistemas nacionais de saúde, a preparação para a identificação e resposta a emergências sanitárias também tem tido destaque na agenda, com ênfase em sistemas de informação, monitoramento e alerta, vigilância e identificação de patógenos, fortalecimento de sistemas laboratoriais e, em alguns casos, na questão das vacinas. Além disso, as análises e proposições organizaram-se fortemente em torno da “resiliência” desses sistemas, com concepções variadas sobre o significado do termo e suas implicações metodológicas, práticas e políticas ^3^. No entanto, mesmo definições abrangentes de resiliência parecem insuficientes para dar conta dos problemas evidenciados, muitos dos quais antecedem o cenário de crise, extrapolam o âmbito das emergências e ultrapassam as fronteiras dos países.

A América Latina foi duramente atingida pela crise multidimensional decorrente da COVID-19, com profundas repercussões econômicas, sociais e sanitárias ^4^. A região expressa a complexa interação entre fatores estruturais, institucionais e políticos nos caminhos das respostas dos países e nos trágicos resultados registrados. As desigualdades estruturais, associadas a economias dependentes, à alta informalidade laboral e a sistemas de proteção social frágeis, representaram um contexto adverso para lidar com uma crise complexa.

Os sistemas de saúde latino-americanos, em sua maior parte, historicamente caracterizaram-se pela segmentação de clientelas, pela fragmentação organizacional, pelo subfinanciamento público e por subsídios estatais ao setor privado, que tende a se concentrar nas capitais e em áreas economicamente dinâmicas. Nas últimas décadas, diversos países implementaram reformas em saúde, com diferentes sentidos, abrangências e temporalidades. Em alguns casos, houve alterações na configuração institucional dos sistemas de saúde, sem que isso superasse suas contradições estruturais.

A Reforma Sanitária no Brasil nos anos 1980, no contexto da democratização, levou à criação do Sistema Único de Saúde (SUS), público e universal, cuja implantação nas décadas seguintes favoreceu importantes avanços em saúde, ainda que sob financiamento insuficiente e sem colidir com o setor privado no país. Chile e Colômbia adotaram, nas décadas de 1980 e 1990, reformas privatizantes que reiteraram a segmentação e as desigualdades entre grupos sociais, com iniciativas posteriores de mudanças incrementais para atenuar as iniquidades. A Argentina manteve, no período, o arranjo segmentado de base corporativa, com a progressiva intensificação de mecanismos de mercado e expansão ou retração de programas públicos específicos, a depender da orientação governamental. O México, sem romper com o arranjo corporativo predominante, adotou um Seguro Popular de Saúde entre 2000 e 2018 para ampliar a cobertura aos trabalhadores informais e pobres, com resultados questionáveis. A mudança de governo alterou os rumos das estratégias de universalização e, em 2026, anunciou-se a criação de um Serviço Universal de Saúde.

Tais sistemas, que já enfrentavam dificuldades para atender às necessidades de saúde diante de transformações demográficas, epidemiológicas e sociais, tiveram dificuldades para lidar com a pandemia de COVID-19 de forma coordenada, devido a limitações internas e a questões globais. As assimetrias mundiais no desenvolvimento científico e tecnológico, na produção e no acesso a insumos estratégicos (como vacinas) e a dependência de importações exacerbaram a vulnerabilidade das nações latino-americanas. Durante a pandemia, os países buscaram soluções variadas, que envolveram negociações bilaterais e com a indústria farmacêutica transnacional para a compra de insumos e, em poucos casos, como no brasileiro, a transferência de tecnologias. 

Em alguns países, o cenário político também foi adverso: governos neoliberais, conservadores e antidemocráticos fragilizaram as instituições públicas e prejudicaram sua capacidade de resposta, por meio da omissão, da negação da ciência ou do escasso diálogo social. A experiência dos países latino-americanos das últimas décadas demonstra que a política e a orientação governamental importam tanto em períodos de relativa estabilidade quanto em momentos de crise. 

Abordar os desafios dos sistemas de saúde pós-pandemia na América Latina, portanto, exige mais do que a preparação para responder a emergências sanitárias - sem dúvida, uma função essencial dos sistemas - ou mesmo à resiliência dos sistemas de saúde. Requer a compreensão dos problemas estruturais desses sistemas e reformas capazes de enfrentá-los, no âmbito do financiamento, da ciência e tecnologia, do Complexo Econômico-Industrial da Saúde (CEIS), das relações público-privadas, da gestão do trabalho, da formação dos profissionais e dos modelos de atenção à saúde. Isso inclui o fortalecimento da atenção primária à saúde e da vigilância em saúde, de forma contínua, com capacidades relacionadas à identificação e à resposta a emergências. Exige também ousadia nas estratégias de universalização, buscando superar as barreiras associadas à inserção laboral ou à capacidade de pagamento por serviços e construir bases institucionais sólidas para o acesso universal. Também é fundamental a articulação com outras políticas públicas, na perspectiva da determinação social dos processos saúde-doença, associada ao conhecimento epidemiológico e das dinâmicas sociais. 

Ou seja, sistemas de saúde públicos bem estruturados, financiados e integrados, com solidez institucional em suas diversas dimensões, estarão mais preparados para atender às necessidades de saúde cotidianas das populações e para identificar e responder às emergências sanitárias de forma mais oportuna e adequada.

Finalmente, o enfrentamento desses desafios, além de sua dimensão técnico-científica, envolve também uma dimensão política, tanto internacional quanto nacional. No cenário global, a ascensão de governos de extrema direita, a intensificação de conflitos e as mudanças geopolíticas recentes - incluindo a saída dos Estados Unidos da Organização Mundial da Saúde ^5^ ^,^ ^6^ - afetam o multilateralismo e incidem sobre os países ^7^. Na América Latina, há necessidade de reforçar, na política externa, a cooperação internacional e a integração regional ^8^, bem como de promover outras iniciativas de articulação no Sul Global para aumentar a soberania sanitária ^9^. Em alguns países da região, situações de instabilidade econômica e política geram preocupação, assim como governos neoliberais, conservadores e antidemocráticos, que ameaçam direitos sociais duramente conquistados e obstaculizam avanços na saúde. Porém, é importante que os diferentes grupos das sociedades estejam atentos às iniciativas dos governos na região. Qualquer mudança de governo, independentemente de sua ideologia ou agenda, não deve transgredir os direitos já adquiridos: é um dever ético reconhecer os avanços, assim como criticar os retrocessos.

Em um cenário complexo, é fundamental realizar pesquisas e refletir criticamente sobre o papel das instituições e da agência política na reorientação de rumos e na construção de sociedades mais igualitárias na América Latina. A região, historicamente, foi um espaço de exploração, de violências e de injustiças, mas também de resistências, lutas e reconstruções. A expansão de direitos, inclusive na área da saúde, sempre envolveu a mobilização de diversos atores sociais, muitas vezes em contextos adversos. Os sistemas nacionais de saúde, se fortalecidos em seu caráter público e universal, podem fazer muito mais do que responder a emergências: como pilares do desenvolvimento inclusivo, da proteção social, do trabalho qualificado e da participação democrática, oferecem amplas possibilidades de ação prática, com imenso potencial de transformação social e de impacto nas condições de vida dos povos.

Ao reunir análises sobre diferentes trajetórias e questões estratégicas a serem enfrentadas pelos sistemas de saúde na região, o Suplemento *Desafios dos Sistemas de Saúde na América Latina Pós-Pandemia* de *Cadernos de Saúde Pública* amplia e concretiza a agenda proposta neste editorial, oferecendo elementos para pensar caminhos de fortalecimento institucional, cooperação regional e afirmação do direito à saúde. Suas contribuições, portanto, não apenas aprofundam o debate, mas também ajudam a sustentar uma perspectiva de transformação dos sistemas de saúde orientada pela equidade, pela universalidade e pela soberania sanitária.

# Translation: Desafíos de los sistemas de salud en América Latina: más allá de las emergencias

**DOI:** 10.1590/0102-311XES112826

**Authors:** Cristiani Vieira Machado, Mónica Uribe-Gómez, Alex Alarcón Hein

**Affiliations:** 1 Escola Nacional de Saúde Pública Sergio Arouca, Fundação Oswaldo Cruz, Rio de Janeiro, Brasil.; 2 Facultad de Ciencias Humanas y Económicas, Universidad Nacional de Colombia, Medellín, Colombia.; 3 Escuela de Salud Publica Salvador Allende, Universidad de Chile, Santiago, Chile.

La pandemia de COVID-19 ha sido una de las mayores emergencias sanitarias jamás registradas, con más de 7 millones de muertes confirmadas en todo el mundo y estimaciones de unos 20 millones de muertes totales causadas por la emergencia, teniendo en cuenta la subnotificación de casos y el exceso de mortalidad general. A esto se suma el impacto negativo en la atención de otras enfermedades, debido a la sobrecarga del sistema de salud, lo que generó muertes por diversas causas y consecuencias a mediano y largo plazo. La crisis multidimensional asociada a la emergencia sanitaria fue brutal, lo cual muestra la relación perversa entre la pandemia y las desigualdades globales e internas en los países ^1^.

Seis años después del inicio de la pandemia, persisten incertidumbres y desafíos en las esferas del conocimiento científico y las políticas de salud, en un escenario marcado por cambios en la geopolítica global, por amenazas a la soberanía de los países y por retrocesos en los derechos sociales y humanos.

La pandemia señaló los límites del multilateralismo, de los mecanismos de integración entre países y de las respuestas de los sistemas nacionales de salud a nivel mundial, lo cual requiere cambios profundos en varios ámbitos. De manera global, impulsó el debate sobre la preparación para futuras emergencias sanitarias y condujo a la conclusión del Acuerdo sobre Pandemias, después de más de tres años de negociaciones, cuya implementación depende de normas complementarias ^2^. 

Respecto a los sistemas nacionales de salud, la agenda destaca la preparación para la identificación y la respuesta ante emergencias sanitarias, con especial énfasis en los sistemas de información, monitoreo y alerta, vigilancia e identificación de patógenos, fortalecimiento de los sistemas de laboratorio y, en algunos casos, el tema de vacunas. Además, los análisis y las propuestas se organizaron fuertemente en torno a la “resiliencia” de estos sistemas, con concepciones variadas sobre el significado del término y sus implicaciones metodológicas, prácticas y políticas ^3^. Sin embargo, incluso las definiciones más amplias de resiliencia parecen insuficientes para dar cuenta de los problemas que se evidencian, muchos de los cuales son anteriores al escenario de crisis, trascienden el ámbito de las emergencias y traspasan las fronteras de los países.

América Latina fue afectada duramente por la crisis multidimensional derivada de la COVID-19, con profundas repercusiones económicas, sociales y sanitarias ^4^. La región manifiesta la compleja interacción entre factores estructurales, institucionales y políticos en los caminos de la respuesta de los países y en los trágicos resultados registrados. Las desigualdades estructurales, asociadas a la economías dependientes, a la alta informalidad laboral y a frágiles sistemas de protección social, representaron un contexto adverso para enfrentar una crisis compleja.

La mayor parte de los sistemas de salud latinoamericanos se caracteriza históricamente por la segmentación de la población atendida, la fragmentación organizacional, la insuficiente financiación de fondos públicos y de subsidios estatales al sector privado, que tiende a centrarse en las capitales y las áreas económicamente dinámicas. Varios países han implementado reformas sanitarias en las últimas décadas, con diferentes significados, alcances y temporalidades. En algunos casos, hubo cambios en la configuración institucional de los sistemas de salud, sin que esto superara sus contradicciones estructurales.

La Reforma Sanitaria en Brasil en los años 1980, en el contexto de la democratización, llevó a la creación del Sistema Único de Salud (SUS), público y universal, cuya implementación en las décadas siguientes contribuyó con importantes avances en salud, aunque con financiamiento insuficiente y sin impactar el sector privado en el país. En las décadas de 1980 y 1990, Chile y Colombia avanzaron en reformas privatizadoras que reforzaron la segmentación de sus sistemas de salud y ampliaron las desigualdades entre grupos poblacionales, aunque posteriormente introdujeron reformas incrementales destinadas a mitigar parcialmente estos efectos. Argentina mantuvo en el período el acuerdo corporativo segmentado, con la intensificación progresiva de los mecanismos de mercado y la expansión o reducción de programas públicos específicos a depender de la orientación del gobierno. Sin romper con el acuerdo corporativo, México adoptó el Seguro Popular de Salud entre 2000 y 2018 para ampliar la cobertura a los trabajadores informales y pobres, con cuestionables resultados. El cambio de gobierno modificó el rumbo de las estrategias de universalización; y en 2026 se anunció la creación de un Servicio Universal de Salud.

Dichos sistemas, que ya enfrentaban dificultades para satisfacer las necesidades de salud ante las transformaciones demográficas, epidemiológicas y sociales, tuvieron dificultades para coordinar la respuesta a la pandemia de COVID-19 de manera coordinada, debido a limitaciones internas y problemas globales. Las asimetrías globales en el desarrollo científico y tecnológico, en la producción y en el acceso a insumos estratégicos (como las vacunas) y la dependencia de las importaciones acentúan la vulnerabilidad de las naciones latinoamericanas. Durante la pandemia, los países buscaron soluciones variadas, que implicaron negociaciones bilaterales y con la industria farmacéutica transnacional para la compra de insumos y, en algunos casos como en Brasil, la transferencia de tecnologías. 

En algunos países, el escenario político también fue adverso: los gobiernos neoliberales, conservadores y antidemocráticos debilitaron las instituciones públicas y socavaron su capacidad de respuesta mediante la omisión, la negación de la ciencia o el escaso diálogo social. La experiencia de los países latinoamericanos en las últimas décadas muestra que la política y la orientación del gobierno son importantes tanto en períodos de relativa estabilidad como en tiempos de crisis. 

Por lo tanto, abordar los desafíos de los sistemas de salud en la pospandemia en América Latina requiere algo más que la preparación para responder a las emergencias sanitarias, sin duda una función central de los sistemas, o incluso la resiliencia de los sistemas de salud. Requiere comprender los problemas estructurales de estos sistemas y reformas capaces de enfrentarlos en el ámbito de la financiación, la ciencia y la tecnología, del Complejo Económico-Industrial de la Salud (CEIS), de las relaciones público-privadas, de la gestión del trabajo, de la formación de profesionales y de los modelos de atención em saluda. Esto incluye fortalecer la atención primaria de salud y la vigilancia sanitaria de manera continua con capacidades relacionadas con la identificación y respuesta a emergencias. También requiere audacia en las estrategias de universalización con el fin de superar las barreras asociadas con la inserción laboral o la capacidad de pagar por los servicios y construir bases institucionales sólidas para el acceso universal. Además, es fundamental la articulación con otras políticas públicas desde la perspectiva de la determinación social de los procesos salud-enfermedad, asociada al conocimiento epidemiológico y a las dinámicas sociales. 

En otras palabras, los sistemas de salud pública bien estructurados, financiados e integrados, con solidez institucional en sus diversos ámbitos, estarán mejor preparados para atender las necesidades diarias de salud de las poblaciones e identificar y responder a las emergencias sanitarias de manera más oportuna y adecuada.

Finalmente, el afrontamiento de estos desafíos, además de su dimensión técnico-científica, implica también una dimensión política tanto internacional como nacional. En el ámbito mundial, el ascenso de los gobiernos de extrema derecha, la intensificación de los conflictos y los recientes cambios geopolíticos, incluida la retirada de los Estados Unidos de la Organización Mundial de la Salud ^5^ ^,^ ^6^, afectan al multilateralismo e impactan los países ^7^. En América Latina, es necesario fortalecer la cooperación internacional y la integración regional en política exterior ^8^, así como promover otras iniciativas de articulación en el Sur Global para aumentar la soberanía sanitaria ^9^. En algunos países de la región, las situaciones de inestabilidad económica y política generan preocupación, así como gobiernos neoliberales, conservadores y antidemocráticos, que amenazan los derechos sociales conquistados y obstaculizan los avances en materia de salud. Sin embargo, es importante que los diferentes grupos de la sociedad estén atentos a las iniciativas de los gobiernos de la región. Cualquier cambio de gobierno, independientemente de su ideología o programa, no debe vulnerar los derechos ya adquiridos: es un deber ético reconocer los avances, así como criticar los retrocesos.

En un contexto complejo, es esencial llevar a cabo estudios y reflexionar críticamente sobre el papel de las instituciones y los actores políticos en la reorientación de los rumbos y en la construcción de sociedades más igualitarias en América Latina. La región, históricamente, ha sido un espacio de explotación, violencia e injusticia, y también de resistencia, luchas y reconstrucciones. La expansión de los derechos, incluso en el campo de la salud, siempre implica la movilización de diversos actores sociales, muchas veces en contextos adversos. Los sistemas nacionales de salud, si se fortalecen en su carácter público y universal, pueden hacer mucho más que responder a las emergencias: como pilares del desarrollo inclusivo, de la protección social, del trabajo calificado y de la participación democrática, ofrecen amplias posibilidades de acción práctica, con un inmenso potencial de transformación social e impacto en las condiciones de vida de los pueblos.

Al reunir análisis sobre diferentes trayectorias y temas estratégicos que serán enfrentados por los sistemas de salud en la región, el Suplemento *Desafíos de los Sistemas de Salud en América Latina tras la Pandemia* de *Cadernos de Saúde Pública* amplía y concreta el programa propuesto en este editorial al ofrecer elementos para pensar en formas de fortalecimiento institucional, cooperación regional y afirmación del derecho a la salud. Sus aportes, por lo tanto, no solo profundizan el debate, sino que también ayudan a sostener una perspectiva de transformación de los sistemas de salud guiada por la equidad, la universalidad y la soberanía sanitaria.
